# Long-distance RNA–RNA interactions shape dynamic RNA architectures and functional states in plant positive-strand RNA viruses

**DOI:** 10.3389/fmicb.2026.1957771

**Published:** 2026-09-30

**Authors:** Deya Wang, Haoran Wu, Xianping Zhang, Qingle Chang, Xuefeng Yuan

**Affiliations:** 1 College of Life Sciences, Zaozhuang University, Zaozhuang, China; 2 Shandong Provincial Key Laboratory of Agricultural Microbiology, Department of Plant Pathology, College of Plant Protection, Shandong Agricultural University, Tai’an, China

**Keywords:** dynamic RNA structure, gene expression and replication, long-distance RNA–RNA interactions (LDRIs), plant positive-strand RNA viruses, RNA structural remodeling

## Abstract

The genomes of positive-strand RNA [(+)ssRNA] viruses function as dynamic RNA molecules that participate in multiple, sometimes competing, processes during infection, including translation, genome replication, subgenomic RNA (sgRNA) transcription, and genome packaging. These distinct processes require viral RNAs to adopt different conformational states, which are influenced by RNA–RNA interactions and associated viral or host factors. Therefore, understanding how viral RNAs undergo structural remodeling in space and time, and how they transition between different functional states, represents a central question in the regulation of positive-strand RNA virus gene expression. Recent studies of diverse plant positive-strand RNA viruses have revealed that long-distance RNA–RNA interactions (LDRIs) serve as important regulatory mechanisms that shape viral RNA higher-order structures and functional states. This review summarizes recent advances in understanding how LDRI-mediated RNA conformational transitions contribute to the formation of higher-order viral RNA structures and support diverse viral RNA functions. We propose that LDRIs should be viewed not only as physical connections between distant RNA regions, but also as dynamic structural elements embedded within viral RNA folding landscapes, where they contribute to transitions between alternative RNA conformations and functional states.

## Introduction

Positive-sense single-stranded RNA [(+)ssRNA] viruses represent approximately 70% of recognized plant virus genera and cause devastating diseases in agricultural crops, posing substantial threats to global food security (Mifsud et al., 2022; Tatineni and Hein, 2023; Iqbal et al., 2026). Unlike most cellular mRNAs that primarily serve as templates for protein synthesis, plant (+)ssRNA virus genomes function not only as carriers of genetic information but also as highly dynamic functional RNA molecules (Watters et al., 2018; Geng et al., 2021; Zhang et al., 2025). During infection, viral RNAs must accommodate diverse functional requirements, including protein translation, genome replication, subgenomic RNA (sgRNA) synthesis, and genome packaging (Annamalai and Rao, 2006; Komoda et al., 2007; Ye et al., 2021; Jaafar and Kieft, 2019). Because these processes rely on the reconuration of the same RNA sequence space and structural states, they are not necessarily separated into strictly ordered stages but are dynamically coordinated throughout the viral life cycle (Lozano and Martínez-Salas, 2015; Nalewaj and Szabat, 2022; Hill and Brierley, 2023). Therefore, viral RNAs must undergo precise structural remodeling to adopt distinct higher-order conformations that support specific biological functions. Increasing evidence indicates that cis-acting RNA elements, long-range RNA conformational rearrangements, and interactions with viral or host factors collectively shape these dynamic RNA architectures and regulate functional transitions during infection (Watters et al., 2018; Hyodo et al., 2019; Geng et al., 2020; Chkuaseli and White, 2022; den Boon et al., 2024).

Long-distance RNA–RNA interactions (LDRIs) provide an important mechanism for coordinating diverse viral RNA functions (Figure 1). They include direct base-pairing interactions between distant regions within the same RNA molecule as well as intermolecular interactions between separate viral RNAs (Chkuaseli and White, 2018, 2023; Newburn and White, 2019). By bringing spatially separated RNA elements into proximity, LDRIs can promote the formation of higher-order RNA architectures, regulate accessibility of functional RNA motifs, and influence transitions between alternative RNA conformational states (Miras et al., 2017; Watters et al., 2018; Ganser et al., 2019).

**FIGURE 1:**
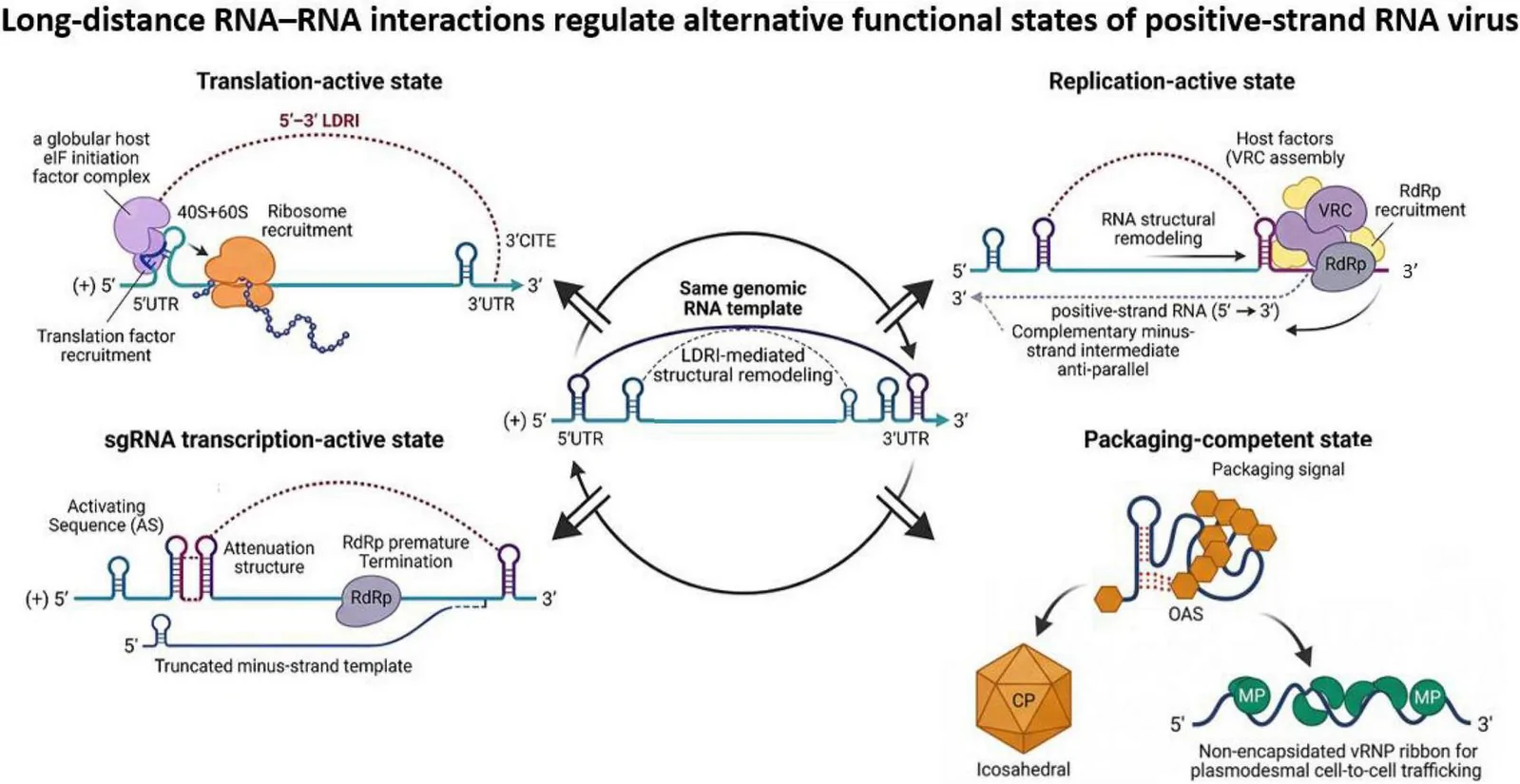
Long-distance RNA–RNA interactions regulate alternative functional states of positive-strand RNA virus genomes. Translation: LDRIs between the 5′untranslated region (5′UTR) and distal translation regulatory elements, including 3′cap-independent translation elements (3′CITEs), promote genome circularization and facilitate recruitment of translation initiation factors and ribosomes to the 5′ end, thereby enhancing viral protein synthesis. Genome replication: During replication, LDRIs juxtapose spatially separated replication-associated RNA elements to establish a functional RNA scaffold for viral replicase complex assembly. This RNA architecture facilitates recruitment of viral RNA-dependent RNA polymerase (RdRp) and initiation of complementary-strand synthesis. Subgenomic RNA transcription: LDRI-mediated interactions between distant regulatory RNA elements generate attenuation structures that control premature termination of viral polymerase during minus-strand synthesis. These RNA conformations regulate the production of subgenomic RNA (sgRNA) and may involve hierarchical RNA folding processes mediated by auxiliary long-range interactions. Genome packaging: During genome packaging, alternative LDRIs promote RNA compaction and higher-order genome organization, facilitating the formation of packaging-competent RNA conformations. These structural rearrangements enhance exposure or assembly of packaging determinants, promote capsid protein recruitment, and support selective encapsidation.

Previous studies established the widespread occurrence and functional diversity of LDRIs in plant positive-strand RNA viruses. Chkuaseli and White (2018) provided an important framework by summarizing major classes of intragenomic LDRIs and their roles in viral RNA regulation, while Newburn and White (2019) highlighted intermolecular RNA–RNA interactions in segmented RNA viruses. However, subsequent studies have expanded this concept from individual RNA contacts toward dynamic structural regulation. Recent work has demonstrated that LDRIs are embedded within complex RNA folding landscapes, where alternative and cooperative interactions reshape viral RNA architectures and determine distinct functional states.

Several advances after these earlier reviews have particularly broadened this field, with representative developments summarized in Table 1. Structural analyses of the Wheat yellow mosaic virus (WYMV) IRES revealed that long-range interactions contribute to dynamic conformational equilibria associated with cap-independent translation (Geng et al., 2020). Studies of RCNMV further showed that local RNA folding can regulate the accessibility of intermolecular interaction sites, demonstrating that LDRI formation depends on the structural context of interacting elements (Im et al., 2021). In PEMV1, LDRI-mediated remodeling of the PRTE–DRTE region provided a model in which ribosome-driven RNA rearrangement exposes hidden interaction motifs to establish a readthrough-active structure (Chkuaseli and White, 2022). In addition, studies of PEMV2 and class 2 umbravirus-like RNAs revealed that intermolecular RNA interactions and competing long-range contacts can generate alternative transcriptional states (Chkuaseli and White, 2023; Mikkelsen et al., 2023). These findings support an emerging view that LDRIs function not simply as static connections between distant RNA regions, but as dynamic structural regulators that enable viral RNAs to transition between alternative functional architectures. In this review, we summarize recent advances in understanding how LDRI-mediated RNA structural remodeling shapes dynamic RNA architectures and regulates functional states of plant positive-strand virus.

**TABLE 1:** Representative LDRI-mediated RNA structural remodeling events in plant positive-strand RNA viruses.

Virus (family/genus)	LDRI elements	Interaction type and distance	Biological process	Dynamic conformational mechanism & structural transitions	Key references
Tomato bushy stunt virus (TBSV) (Tombusvirus/ Tombusviridae) (+)ssRNA (∼4.8 kb)	UL/DL, AS1/RS1, AS2/RS2, DE/CE, 5′UTR–3′CITE)	Intramolecular; kb-scale genome-wide RNA network	Translation, replication, and sgRNA synthesis	Multiple cooperative LDRIs assemble a hierarchical RNA folding pathway, in which sequential structural rearrangements generate alternative RNA architectures required for translation, replication, and sgRNA synthesis.	Chkuaseli and White, 2018, 2020
Carnation Italian ringspot virus (CIRV) (Tombusviridae; Tombusvirus)	RTSL–DRTE/PRTE; RTSL-TL–SL59; AS1/RS1; AS1-SL3′/SL59-3′	Intramolecular; kb-scale interactions within the viral genome	Translational readthrough, sgRNA1 transcription, and genome replication regulation	A hierarchical RNA folding pathway involving sequential LDRIs remodels LD2 into an RdRp attenuation structure, demonstrating that LDRIs function as dynamic architectural regulators rather than isolated RNA bridges.	Chkuaseli and White, 2020, 2023
Pea enation mosaic virus 1 (PEMV1) Enamovirus; Tombusviridae	PRTE ↔ DRTE	Intramolecular; hundreds-nt scale	Stop-codon readthrough	Ribosome-mediated remodeling exposes complementary RNA motifs, enabling long-range pairing and regulating readthrough efficiency.	Chkuaseli and White, 2022
Class 2 Umbravirus-like RNAs (Tombusviridae-related)	5′ proximal loop ↔ 3′ FSE core loop	Intramolecular (∼2.5 kb)	−1 PRF dynamic switching	Multiple competing long-distance interactions toggle the frameshift element between active and inactive conformers.	Mikkelsen et al., 2023
Wheat yellow mosaic virus (WYMV)	5′–3′ end pairing & IRES domains	Intramolecular	IRES-mediated translation	Modulates dynamic tertiary conformational equilibrium among H1, H2, and LR1 and facilitates eIF4E recruitment.	Geng et al., 2020

## LDRI-mediated RNA structural dynamics shape viral RNA functional states

Long-distance RNA–RNA interactions (LDRIs) provide a structural framework for reshaping viral RNA architectures and establishing functional RNA states. Rather than acting as isolated contacts between distant RNA regions, LDRIs are frequently integrated into hierarchical RNA folding pathways, where they regulate the accessibility of functional motifs, stabilize higher-order RNA structures, and facilitate transitions between alternative conformations. Recent studies of plant positive-strand RNA viruses have revealed diverse modes of LDRI-mediated structural remodeling, ranging from local motif exposure and conformational tuning to stepwise assembly of complex RNA architectures.

A representative example of LDRI-mediated structural remodeling is provided by translational readthrough regulation in Pea enation mosaic virus 1 (PEMV1) (Demler et al., 1997; Liu et al., 2009; Chkuaseli and White, 2022). In PEMV1, a proximal readthrough element (PRTE) located near the coat protein (CP) stop codon interacts with a distal readthrough element (DRTE) positioned several hundred nucleotides downstream. In the proposed inactive conformation, the CCCC motif required for PRTE–DRTE kissing-loop interaction is sequestered within the stem of SL1, preventing productive long-range pairing. Ribosome progression through the upstream coding region is proposed to remodel the local RNA structure, allowing SL1 to refold into SL2 and exposing the CCCC motif within the apical loop. This newly accessible sequence can subsequently pair with the complementary GGGG motif in SL4 of the DRTE, generating a higher-order RNA structure that promotes stop-codon readthrough and CP-RTD synthesis. Thus, PEMV1 illustrates how LDRIs can regulate viral translation by controlling the structural accessibility of regulatory RNA motifs through conformational masking and remodeling (Figure 2A).

**FIGURE 2:**
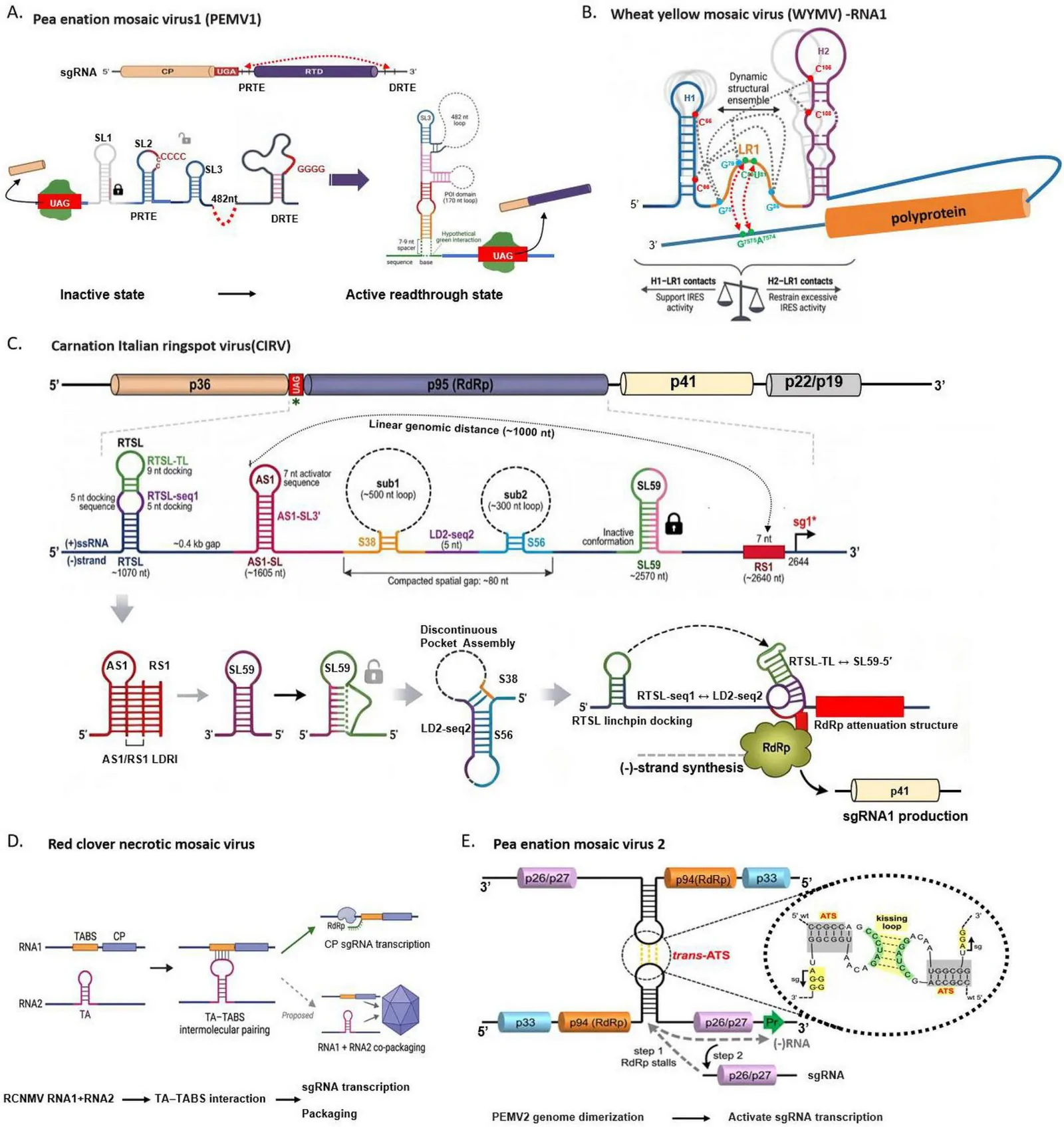
Long-distance RNA–RNA interaction (LDRI)-mediated RNA structural remodeling and functional state transitions in plant positive-strand RNA viruses. **(A)** PEMV1 readthrough: Translating 80S ribosomes stalling at the CP UGA stop codon mechanically unwind local stem-loop 1 (SL1; 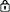 to 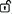), exposing the CCCC motif in SL2 to form a long-range kissing loop with distal DRTE (SL4 GGGG) across an intervening 482 nt loop, driving extended CP-RTD synthesis. **(B)** WYMV RNA1 5′ IRES: The 5′ UTR IRES recruits host eIF4E via hairpin H2. Long-range 5′–3′ bridging modulates an ensemble of tertiary conformers, acting as a balance beam to support translation while restraining excessive IRES activity. **(C)** CIRV sgRNA1 transcription: Subdomains sub1 and sub2 condense the linear ∼1000 nt AS1–RS1 distance to an ∼80 nt spatial gap. Ground-state SL59 remains auto-inhibited (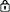). Base-pairing between AS1 and RS1 prompts AS1-SL3′ to competitively melt SL59, unmasking the docking sequence (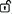) to assemble a bipartite pocket across S56 where distal RTSL (∼1.5 kb upstream) docks as a linchpin roadblock to arrest transcribing RdRp and trigger sgRNA1 transcription. **(D)** RCNMV segment coordination: Intermolecular pairing between RNA2 TA and unmasked RNA1 TABS bifurcates into two distinct biological outputs: arresting minus-strand synthesis to activate CP sgRNA transcription (top arrow), and assembling a quaternary Origin of Assembly (OAS) to direct RNA1 + RNA2 virion co-packaging (bottom arrow). **(E)** PEMV2 homodimerization: Monopartite PEMV2 forms an obligate co-infection complex with PEMV1 (providing movement functions p26/p27). In contrast to tombusvirus intramolecular folding, PEMV2 governs transcription attenuation via concentration-dependent kissing-loop homodimerization (trans-ATS, 5′-GGCGCC-3′) between two identical genomes, physically stalling transcribing RdRps. *Denotes the stop codon UAG.

Beyond simple motif exposure, LDRIs can also regulate viral functions by modulating dynamic RNA conformational equilibria. In Wheat yellow mosaic virus (WYMV) RNA1, the 5′ untranslated region contains an internal ribosome entry site (IRES) composed of multiple structural domains, including H1, LR1, and H2 (Geng et al., 2020). Rather than adopting a single fixed active structure, the WYMV IRES exists as a dynamic ensemble of alternative conformations. Long-range interactions between distant RNA regions contribute to shaping this structural landscape by influencing the relative stability and organization of IRES-associated domains. These conformational transitions affect the accessibility and arrangement of translation-related elements, thereby supporting efficient cap-independent translation (Figure 2B). This example highlights that LDRIs can function as modulators of RNA structural equilibrium, allowing viral RNAs to fine-tune functional outputs through dynamic conformational selection.

The most elaborate example of LDRI-driven RNA structural remodeling is observed in Carnation Italian ringspot virus (CIRV), where multiple intragenomic interactions cooperate to assemble a functional RNA regulatory architecture (Chkuaseli and White, 2020). Formation of the CIRV transcriptional attenuation structure follows a hierarchical RNA folding pathway. Initial folding of LD2 subdomains brings the distantly located AS1 and RS1 elements into spatial proximity, reducing their separation from approximately 1000 nucleotides in the linear genome to an approximately 80-nucleotide structural distance that facilitates AS1/RS1 base pairing. This interaction stabilizes the basal region of LD2 and promotes subsequent remodeling of neighboring RNA elements. Formation of the AS1-SL3′/SL59-3′ interaction releases previously sequestered SL59-5′ sequences, enabling assembly of a discontinuous docking pocket composed of LD2-seq2 and SL59-5′. The distal RTSL element is subsequently recruited into this pocket through cooperative interactions, generating a higher-order RNA structure that attenuates viral RdRp progression and activates sgRNA1 transcription (Figure 2C). Unlike PEMV1 and WYMV, where LDRIs primarily regulate local RNA accessibility or conformational balance, CIRV demonstrates that multiple LDRIs can act cooperatively as an RNA folding pathway to transform a flexible viral genome into a functional regulatory machine.

These examples reveal distinct but interconnected principles of LDRI-mediated RNA regulation. In PEMV1, LDRIs facilitate functional activation through structural unmasking of regulatory motifs; in WYMV, LDRIs reshape the conformational landscape of translation-related RNA elements; and in CIRV, cooperative LDRIs drive hierarchical assembly of a higher-order RNA architecture. Collectively, these studies support a model in which LDRIs act as dynamic structural regulators that enable viral RNA genomes to transition between functionally distinct states required throughout the viral life cycle.

## Intermolecular long-distance RNA–RNA interactions

While intramolecular LDRIs remodel the conformation of individual viral RNA molecules, intermolecular LDRIs extend this regulatory principle across separate RNA molecules. Through RNA–RNA communication between different genomic segments or between copies of the same viral RNA, intermolecular interactions can generate cooperative RNA architectures that coordinate viral gene expression and genome regulation. Importantly, these interactions are not solely determined by sequence complementarity but are frequently influenced by the structural accessibility and conformational organization of the participating RNA elements. Recent studies have revealed that intermolecular LDRIs function as dynamic structural switches, in which RNA folding and intermolecular recognition are coupled to establish functional viral RNA states.

A representative example is provided by Red clover necrotic mosaic virus (RCNMV), a bipartite positive-strand RNA virus containing RNA1 and RNA2. The trans-activator (TA) element located in RNA2 specifically base-pairs with the trans-activator binding site (TABS) within RNA1, forming an intermolecular RNA complex required for transcription of the RNA1-encoded coat protein subgenomic RNA (sgRNA) (Sit et al., 1998; Guenther et al., 2004; Bhardwaj et al., 2019). This interaction demonstrates how RNA molecules from separate genomic segments can communicate through direct RNA–RNA recognition to regulate viral gene expression.

Importantly, formation of the TA–TABS complex is controlled by the local structural context of the target RNA region. The TABS sequence within RNA1 is partially masked by upstream intramolecular base pairing, which restricts its accessibility to the TA element. Remodeling of this local RNA structure therefore acts as a structural gating mechanism that regulates intermolecular recognition. Mutational disruption of the upstream pairing alters TA–TABS complex formation *in vitro* and affects sgRNA accumulation during infection, demonstrating that local RNA folding can control the efficiency of intermolecular RNA communication (Figure 2D; Guenther et al., 2004; Im et al., 2021).

The TA region has also been implicated in genome assembly, as RNA2-derived fragments containing the TA sequence can promote packaging of RNA1 (Basnayake et al., 2009). However, whether TA–TABS pairing directly participates in virion assembly remains unresolved. It has not been demonstrated that coat protein recognizes a preassembled TA–TABS duplex during infection or that a transcriptional RNA complex is directly converted into a packaging complex. Therefore, the proposed role of TA-mediated interactions in coordinating RNA recognition and genome packaging should be considered a supported but incomplete mechanistic model.

Whereas RCNMV relies on heterotypic interactions between distinct genomic segments, Pea enation mosaic virus 2 (PEMV2) provides an example of intermolecular LDRIs between identical viral RNA molecules. Unlike the intramolecular attenuation structures observed in related tombusviruses, PEMV2 requires intermolecular genome dimerization to assemble a functional trans-attenuation structure (trans-ATS) (Chkuaseli and White, 2023). Each PEMV2 genome contains an ATS stem–loop carrying a self-complementary apical palindrome. Pairing between the apical loops of two RNA molecules forms a kissing-loop interaction that brings the genomes together and is essential for subgenomic RNA transcription (Figure 2E; Chkuaseli and White, 2023).

Notably, genome dimerization alone is insufficient to define transcriptional activity. Although the kissing-loop interaction does not require extension into a longer duplex, alterations in ATS stem length can disrupt subgenomic RNA transcription without substantially affecting dimer formation *in vitro*. These findings indicate that trans-ATS activity depends not only on intermolecular pairing itself but also on the precise three-dimensional arrangement of the assembled RNA architecture, including stem geometry and the spatial positioning of the regulatory structure relative to the viral RNA-dependent RNA polymerase.

Because intermolecular dimerization requires interactions between multiple RNA molecules, it has been proposed that trans-ATS formation may be influenced by viral RNA abundance during infection, potentially linking RNA accumulation with temporal regulation of subgenomic RNA production. However, direct measurements connecting RNA concentration, dimerization dynamics, and transcriptional output remain unavailable. Thus, PEMV2 provides compelling evidence that intermolecular LDRIs can generate functional RNA regulatory structures, while the mechanisms governing their temporal activation remain to be further clarified.

## Scope and perspective: LDRIs as dynamic RNA architectural networks

Long-distance RNA–RNA interactions (LDRIs) were initially recognized as a mechanism that brings regulatory RNA elements separated in the linear genome into spatial proximity. By enabling communication between distant RNA regions, LDRIs contribute to the formation of functional RNA structures involved in diverse viral processes, including translation regulation, programmed ribosomal frameshifting, stop-codon readthrough, genome replication, and subgenomic RNA (sgRNA) transcription (Miller and White, 2006; Nicholson and White, 2014; Simon and Miller, 2013; Chkuaseli and White, 2018; Newburn and White, 2019). One of the best-characterized examples is provided by 3′ cap-independent translation enhancers (3′CITEs), in which long-distance interactions between the viral 3′ untranslated region (UTR) and the 5′ region establish communication between spatially separated regulatory elements and promote translation initiation (Miller and White, 2006; Nicholson and White, 2014; Simon and Miller, 2013). Different classes of 3′CITEs, including BTE-, PTE-, TSS-, ISS-, and YSS-type elements, illustrate how diverse RNA architectures can achieve functional communication between distant genomic regions (Miller et al., 2007; Gao et al., 2012; Simon and Miller, 2013). In addition, long-distance interactions controlling programmed ribosomal frameshifting, translational readthrough, and sgRNA transcription demonstrate that viral RNA genomes can assemble specialized regulatory structures through interactions among distant RNA elements (Fabian and White, 2004; Chkuaseli and White, 2020, 2022; Mikkelsen et al., 2023).

However, accumulating evidence indicates that the biological significance of LDRIs extends beyond the formation of individual long-range contacts. Viral RNA genomes exist within dynamic folding landscapes in which multiple RNA elements can interact cooperatively or competitively to generate alternative higher-order structures (Wu et al., 2013; Watters et al., 2018). Thus, LDRIs should not be considered merely as static molecular bridges connecting distant sequences, but rather as components of broader RNA architectural networks that contribute to structural transitions among different functional states. The formation and functional consequences of these interactions are influenced by multiple factors, including local RNA folding, sequence accessibility, viral and host RNA-binding proteins, RNA abundance, and the movement of molecular machines such as ribosomes and RNA-dependent RNA polymerases (RdRps) along viral RNA templates (Chkuaseli and White, 2020; Geng et al., 2020). For example, host translation factors, including eIF4E/eIF4G and eIF3, can participate in organizing functional RNA conformations associated with 3′CITE-mediated translation, demonstrating that viral RNA structures are shaped by both intrinsic RNA folding properties and interactions with cellular factors (Liu and Simon, 2022).

Recent studies have expanded the conceptual framework of LDRIs from individual RNA contacts toward dynamic structural remodeling pathways. In Red clover necrotic mosaic virus (RCNMV), local RNA folding controls accessibility of the trans-activator binding site (TABS), thereby regulating intermolecular recognition between RNA1 and RNA2 (Guenther et al., 2004; Im et al., 2021). In Pea enation mosaic virus 1 (PEMV1), the proposed SL1-to-SL2 transition exposes the CCCC motif required for PRTE–DRTE kissing-loop formation, enabling assembly of a readthrough-promoting RNA architecture (Demler et al., 1997; Chkuaseli and White, 2022). Similarly, the Wheat yellow mosaic virus (WYMV) IRES represents a dynamic conformational ensemble in which long-range interactions influence the balance among alternative RNA structures associated with translation regulation (Geng et al., 2020). Beyond these examples, recent analysis of umbravirus-like RNAs demonstrated that multiple competing LDRIs can switch programmed −1 ribosomal frameshifting elements between alternative functional states, providing direct evidence that RNA interaction landscapes can operate as dynamic structural switches (Mikkelsen et al., 2023).

A further advance has been the recognition that LDRIs can participate in hierarchical RNA folding pathways rather than acting as isolated interactions. In tombusviruses, large-scale RNA remodeling involves sequential formation of multiple long-range contacts that progressively organize dispersed RNA elements into functional transcriptional architectures (Chkuaseli and White, 2020). The CIRV attenuation structure represents an example in which successive RNA rearrangements establish a higher-order RdRp regulatory platform, demonstrating that LDRI-mediated folding can occur through ordered structural transitions rather than simple binary pairing events (Chkuaseli and White, 2020). These findings support a model in which viral RNA genomes behave as dynamic structural systems capable of transitioning among multiple conformational states during infection.

Long-distance RNA–RNA interaction-mediated RNA architectures can also arise through interactions between separate viral RNA molecules. In RCNMV, intermolecular pairing between the RNA2 trans-activator (TA) element and the RNA1 trans-activator binding site (TABS) generates a functional RNA complex required for coat protein sgRNA transcription (Sit et al., 1998; Guenther et al., 2004). In Pea enation mosaic virus 2 (PEMV2), an umbravirus within the family Tombusviridae, kissing-loop interactions between two genomic RNA molecules assemble a trans-attenuation structure (trans-ATS) required for sgRNA synthesis (Chkuaseli and White, 2023). These examples extend the concept of viral RNA regulation beyond intramolecular genome folding toward cooperative communication between independent RNA molecules.

The PEMV1/PEMV2 infection system further raises an intriguing possibility for future investigation. PEMV1 and PEMV2 form an obligate synergistic infection complex, in which PEMV1 provides coat protein and aphid transmission functions, whereas PEMV2 contributes movement-related functions (Demler et al., 1997; Ryabov et al., 2001; Chkuaseli and White, 2023). Although direct RNA–RNA interactions between PEMV1 and PEMV2 genomes have not yet been demonstrated, their coordinated replication, packaging, and transmission suggest that cross-genome RNA communication may represent an additional layer of viral RNA regulation. Determining whether inter-viral LDRIs occur during mixed infections, and whether such interactions influence genome organization, packaging, or lifecycle coordination, will provide new insights into the diversity of intermolecular RNA regulatory mechanisms.

Together, these findings support a broader view of plant positive-strand RNA genomes as dynamic structural systems capable of adopting multiple functional conformations. The concept of an RNA architectural network does not imply that all RNA contacts occur simultaneously or remain permanently stable. Instead, it describes a collection of alternative and cooperative RNA interactions that are selectively favored according to structural accessibility, molecular environment, RNA abundance, and the functional requirements of different stages of infection (Figure 3). This framework generates several testable predictions: disruption of one RNA interaction may redistribute the interaction landscape rather than simply abolish RNA regulation, and intermolecular LDRIs may exhibit additional dependence on genome availability and RNA concentration.

**FIGURE 3:**
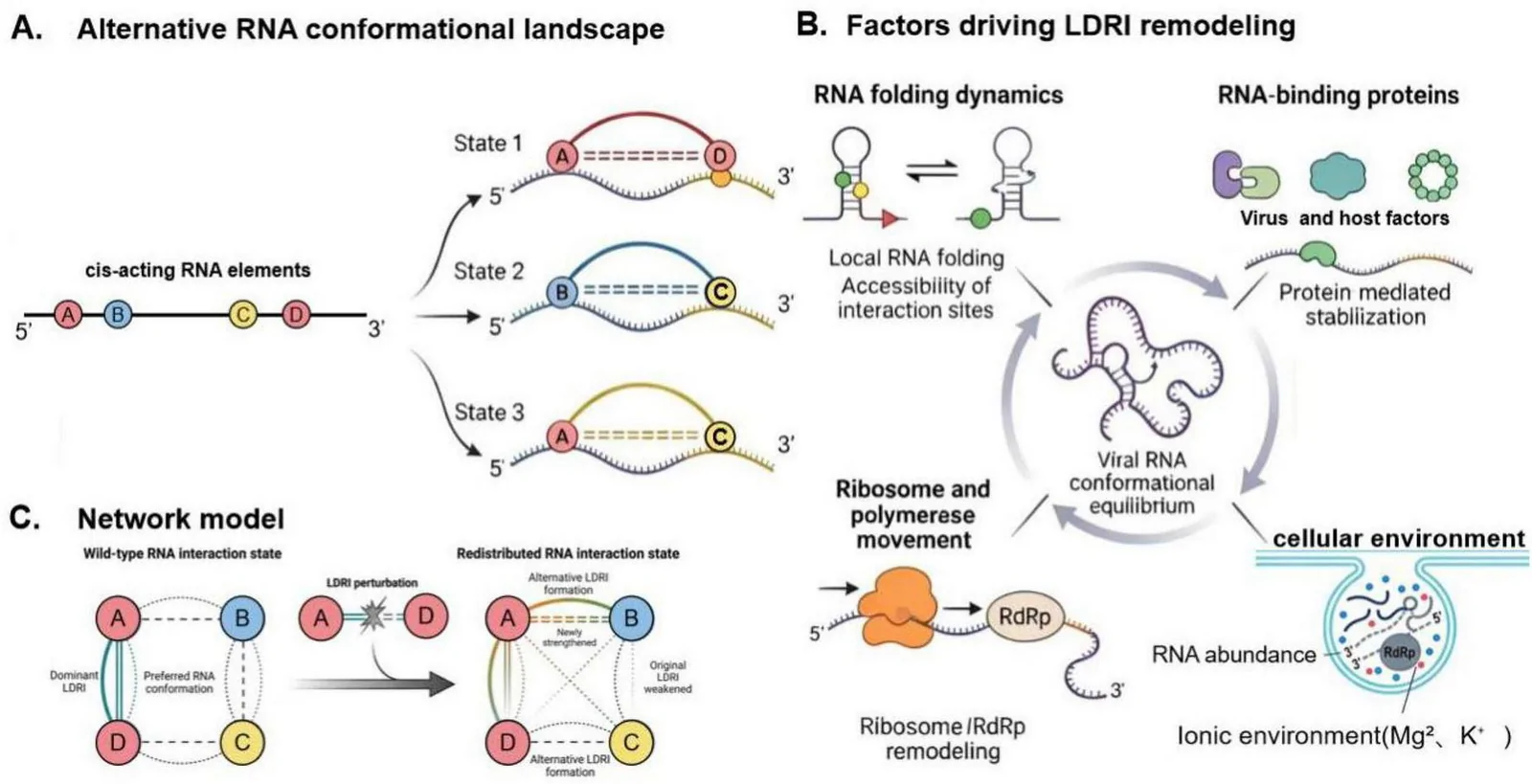
Dynamic remodeling of long-distance RNA–RNA interaction networks underlying viral RNA structural transitions. **(A)** A single viral RNA genome contains multiple regulatory RNA elements that can establish alternative LDRI configurations. Different combinations of RNA–RNA contacts generate distinct higher-order RNA architectures, illustrating the structural plasticity of viral genomes. **(B)** LDRI selection and remodeling are influenced by multiple molecular factors, including local RNA folding dynamics, binding of viral and host RNA-associated proteins, movement of ribosomes or RNA-dependent RNA polymerases along the genome, and RNA abundance. These factors collectively determine the accessibility and stability of competing RNA conformations. **(C)** LDRI networks exhibit dynamic redistribution rather than fixed pairwise interactions. Perturbation of one RNA–RNA interaction does not necessarily abolish RNA regulation but may alter the interaction landscape by weakening one connection and favoring alternative LDRI formation, resulting in a new RNA structural state. The letters A–D indicate different RNA regions predicted to participate in potential long-distance RNA–RNA interactions.

Therefore, understanding LDRI-mediated regulation requires moving beyond the identification of individual RNA contacts toward defining how RNA interaction landscapes are organized, remodeled, and coupled to viral function. The key question is not only which RNA regions interact, but also how alternative LDRI configurations reshape viral RNA architectures and enable transitions between distinct functional states.
